# Application of the combination of CBL teaching method and SEGUE framework to improve the doctor-patient communication skills of resident physicians in otolaryngology department

**DOI:** 10.1186/s12909-024-05185-9

**Published:** 2024-02-27

**Authors:** Nan Zeng, Hui Lu, Shuo Li, Qiong Yang, Fei Liu, Hongguang Pan, Shang Yan

**Affiliations:** 1 Department of Otolaryngology, Huazhong University of Science and Technology Union Shenzhen Hospital (Nanshan Hospital), 518053 Shenzhen, China; 2 Department of Otolaryngology, Shenzhen Children’s Hospital, 7019 Yitian Road, Futian District, 518028 Shenzhen, Guangdong China

**Keywords:** CBL teaching method, SEGUE framework, Department of otolaryngology, Standardized resident training, Doctor-patient communication

## Abstract

**Background:**

To explore the feasibility and effectiveness of applying CBL teaching method and SEGUE Framework in the doctor-patient communication skills of resident physicians in the department of otolaryngology.

**Methods:**

This is an observational study to compare the score changes in doctor-patient communication skills of 120 resident physicians, before and after using CBL combined SEGUE Framework teaching method. The effects of gender, age, grade, educational background and marital status on SEGUE score were analyzed.

**Results:**

Through the combined application of CBL teaching method and SEGUE Framework, the SEGUE score of 120 resident physicians was significantly improved. There was no significant difference in SEGUE score among different sex and marital status of resident physicians. SEGUE score is positively correlated with age; Different grades and educational backgrounds have significant effects on SEGUE score.

**Conclusion:**

The combination of CBL teaching method and SEGUE Framework is feasible and effective in the education program of doctor-patient communication skills for resident physicians in the department of otolaryngology, and worthy of popularization and application in other medical specialties.

## Background

Doctor-patient communication, in form, refers to the bidirectional exchange of information between healthcare professionals and patients and/or their family members during the diagnosis, treatment, and follow-up processes. In essence, it embodies the emotional communication and establishment of trust between the doctor and the patient [1]. In the current biopsychosocial model of medicine, proficient communication skills are essential for building harmonious doctor-patient relationships and addressing clinical issues. The lack of communication between doctors and patients can lead to patient doubts regarding the doctor’s explanation of the condition and treatment plan, as well as increase the difficulty of diagnosis and treatment for doctors. Among the defects in the medical education of our country, one particular issue is that the education on ethics and humanities for medical students is lacking, which would hinder their effective communication with patients and also affect their professional skills and overall qualities.

Otorhinolaryngology is one important medical discipline, but its complexity, diversity, specificity, and limitations in teaching methods and resources, bring several challenges to medical education. To optimize the training quality for students in this discipline, Case-Based Learning (CBL) and SEGUE Framework, two newly emerging tools for medical education, might hold great promise [2–3]. CBL is a teaching approach based on case studies that requires students to independently solve problems and make decisions [2]. In the context of medical education, it plays a crucial role in developing students’ clinical reasoning, problem-solving skills, and the ability to apply theoretical knowledge to real-world medical scenarios. CBL’s theoretical basis in constructivism, problem-based learning, and experiential learning aligns with the core principles of medical education. It offers a pedagogical approach that prepares future healthcare professionals to think critically, solve problems effectively, and provide patient-centered care. While, the SEGUE Framework is an assessment tool to evaluate communication skills, providing a systematic approach to guide physicians in effective doctor-patient communication [3]. As such, the SEGUE Framework is another valuable tool in medical education, helping to foster the development of well-rounded, competent, and compassionate physicians.

Considering the unique characteristic of otorhinolaryngology and the poor current state of medical education in this discipline, it is of great importance to integrate these teaching methods and assess its efficacy. In this study, we aim to explore the effectiveness of integrating CBL with the SEGUE Framework in improving the communication skills of resident physicians in otorhinolaryngology training programs (for short “residency training”).

## Method

### Study participants

We conducted an observational study involving a statistical population currently undergoing normative residency training and selected our sample using a systematic random sampling method. A total of 120 physicians undergoing otorhinolaryngology residency training at 9 training centers in Shenzhen City from January 2022 to June 2022 were selected as the study participants. The inclusion criteria were as follows: ① Second and third-year otorhinolaryngology residents in training; ② First-year residents who had completed at least one month of rotation in the otorhinolaryngology department at their respective training centers. Exclusion criteria included residents who withdrew from the training program for personal reasons. This study was approved by the Ethics Committee of Huazhong University of Science and Technology Union Shenzhen Hospital (Nanshan Hospital). All participating residents who completed the questionnaire were informed and gave their consent for their involvement in the study.

### Research tools

SEGUE Communication Skills Assessment Tool: The SEGUE Framework is a model designed to guide medical students in effective communication with patients. It was developed by Professor Makoul from Northwestern University Feinberg School of Medicine in the United States [4]. In 2006, it was introduced and adapted into Chinese by China Medical University [5]. Research has shown that SEGUE can effectively measure and evaluate the communication skills of Chinese medical students, demonstrating good reliability and validity. SEGUE is an acronym derived from the first letters of several English words, representing the following components: S (Set the Stage): Creating a comfortable environment and establishing trust with patients, making them feel respected and cared for; E (Elicit Information): Guiding patients to actively share their medical history, symptoms, including the duration of illness, location of pain, and nature of symptoms, among others; G (Give Information): Presenting diagnosis results, disease characteristics, treatment plans, and other relevant information to patients in easily understandable language; U (Understand the Patient’s Perspective): Understanding the patient’s perspective on the disease, their attitude towards the treatment plan, and their expectations for treatment outcomes, in order to better serve the patient; E (End the Encounter): Summarizing the discussion, confirming the next steps and action plans with the patient, and ensuring that the patient feels that their concerns have been addressed and attended to. The SEGUE Framework is a structured and systematic communication guide model that helps healthcare professionals communicate effectively with patients and achieve better clinical outcomes. It has been widely used in medical education and clinical practice. Moreover, SEGUE is also a very good assessing tool to evaluate the communication performance [4].

### Research methods

This study commenced in January 2022. Prior to the start of the study, the 120 participating residents received varying levels of theoretical knowledge and practical training in otorhinolaryngology, including departmental education, teaching rounds, and simulated scenarios for medical communication. SEGUE is not only a teaching tool but also an assessing method to evaluate the communication skills. Therefore the SEGUE questionnaire was utilized here to collect data from participants. In January 2022, the first around of survey was conducted using the SEGUE questionnaire. Subsequently, over a six-month period, an expert group composed of teaching and management faculty in otorhinolaryngology from the nine training centers selected cases for CBL based on the requirements outlined in the “Standardized Training for Resident Physicians (2021 Edition) - Otorhinolaryngology Training Guidelines.” These cases were combined with the SEGUE framework and included specific medical communication scenarios for training purposes. In June 2022, the SEGUE questionnaire survey was conducted again. The data from two rounds of surveys based on SEGUE questionnaires were further extracted, summarized and analyzed for comparison.

### Statistical analysis

Data analysis was performed using SPSS 22.0 statistical software. After checking the normality of the metric data, descriptive statistics were presented as ‾X ± *s*, and paired t-tests or analysis of variance (ANOVA) were employed to determine the statistical difference between groups. Pearson correlation analysis was used for assessing correlations. A two-tailed test was conducted, and a *P-*value of < 0.05 was considered statistically significant for detecting differences.

## Results

Through the combination of CBL teaching method and SEGUE framework, the SEGUE scores of 120 residents were significantly improved. The specific content of the score table is analyzed as follows.

### General information and comparison of SEGUE scores among resident physicians

A total of 120 questionnaires were distributed in this study, and all 120 questionnaires were returned, resulting in a response rate of 100%. Overall, 68 males and 52 females were involved, and their age ranged from 23 to 36 years, with an average of 26.13 (± 2.09) years. There were 42 first-year residents, 50 s-year residents, and 28 third-year residents. In terms of education background, 15 residents had a bachelor’s degree, 90 had a master’s degree, and 15 had a doctoral degree. Among the participants, 108 were unmarried and 12 were married. The average SEGUE score prior to training was 77.38 (± 3.78), while the average score improved to 85.91 (± 2.82) after six months of training. A statistically significant difference was observed (*P* < 0.05).

### Comparison of SEGUE scores among resident physicians of different genders

The comparison of SEGUE scores among resident physicians of different genders showed no statistically significant difference (*P* < 0.05). Please refer to Table 1.

**Table 1:** Comparison of SEGUE scores of resident physicians across genders, training years, educational background and marital status

Group	Index	Number	Average score before training	Average score after training	P value
Gender	Male	68	77.28	86.00	0.750
	Female	52	77.50	85.79	
Training Year	First Year	42	75.48	84.98	0.000
	Second Year	50	77.66	85.98	
	Third Year	18	79.71	87.18	
Education background	Undergraduate	15	77.20	85.67	0.000
	Master	90	76.77	85.44	
	Doctor	15	81.20	88.93	
Marital status	Unmarried	108	77.16	85.68	0.053
	Married	12	79.33	88.00	

### Comparison of SEGUE scores of resident physicians across different ages

Pearson correlation analysis revealed a positive correlation between SEGUE scores of resident physicians and their age. The correlation coefficients (r) for the two assessments were 0.338 and 0.333, respectively (*P* < 0.05). Please refer to Table 2.

**Table 2:** Comparison of correlation between SEGUE scores and age among resident physicians of different ages

Group	Pearson Correlation	P value
Before training	0.338**	0.000
After training	0.333**	0.000

### Comparison of SEGUE scores of resident physicians among different training years

Levene’s test revealed unequal variances in SEGUE scores among resident physicians of different training years. The results showed significant differences in SEGUE scores among different training years during the first assessment (*P* < 0.05). After the combined training using CBL methodology and SEGUE Framework, the overall SEGUE scores of the 120 resident physicians significantly improved (*P* < 0.05). However, there were no significant differences in scores between the second and third-year trainees. Please refer to Table 1.

### Comparison of SEGUE scores among resident physicians with different educational backgrounds

Levene’s test revealed heterogeneity of variance in SEGUE scores among resident physicians with different educational backgrounds. One-way analysis of variance (ANOVA) test was used for analyzing the data, and the results showed that after the combined application of CBL teaching method and SEGUE Framework, the SEGUE scores of resident physicians with different educational backgrounds significantly improved (*P* < 0.05). However, there was no significant difference in SEGUE scores between master’s degree and undergraduate resident physicians in the two assessments. Please refer to Table 1.

### Comparison of SEGUE scores among resident physicians with different marital status

The comparison of SEGUE scores among resident physicians with different marital statuses before and after the training showed a significant statistical difference (*P* < 0.05). However, when comparing the scores for each assessment, there was no significant statistical difference observed among resident physicians with different marital statuses. This indicates that marital status does not have a significant statistical impact on SEGUE scores. Please refer to Table 1.

## Discussion

### Current status of doctor-patient communication skills among torhinolaryngology resident physicians

The results of this assessment revealed that the average SEGUE score before training was (77.38 ± 3.78) points, while the score after six months of training was (85.91 ± 2.82) points. The difference between the two scores was statistically significant (*ρ* < 0.05), indicating a significant improvement in scores after the training. Comparing these results with the data on doctor-patient communication skills among resident physicians by Zhu Lingping, Zhu Dan, and others [6–7], it is evident that the otorhinolaryngology resident physicians in the training bases need further enhancement of their doctor-patient communication skills in future training.

Otorhinolaryngology is an independent secondary discipline in clinical medicine. It is highly specialized and involves a wide range of complex course content, encompassing multiple interdisciplinary areas. However, it receives relatively few hours of instruction during undergraduate medical education, and the practical training is not extensive. This lack of in-depth understanding and memorization difficulties pose significant challenges in teaching [8]. Therefore, during the residency training stage, clinical education in otorhinolaryngology faces enormous pressure and emphasizes practical skills and clinical reasoning, ultimately leading to more effective doctor-patient communication.Based on extensive clinical observations, otorhinolaryngology resident physicians often face difficulties in effectively communicating with patients, which can be summarized as follows: ① Frequent use of medical terminology: When communicating with patients, otorhinolaryngology resident physicians may use excessive medical jargon, making it difficult for patients to understand and accept the information [9]. ② Overemphasis on medical condition, neglecting emotional needs of patients: Resident physicians may focus excessively on the medical condition and treatment, overlooking the emotional needs of patients, which can make them feel undervalued. ③ Lack of communication skills: Resident physicians may lack essential communication skills such as active listening, effective expression, appropriate questioning, and providing feedback. This can result in poor communication outcomes and an inability to establish a good doctor-patient relationship [10]. ④ Impatience with patients: Otorhinolaryngology resident physicians may demonstrate impatience with patients who have complex medical conditions, which hinders their ability to effectively explain the diagnosis and treatment, leading to a loss of patient confidence [11]. ⑤ Lack of cultural sensitivity: When communicating with patients, it is important to consider their cultural background and values. Without cultural sensitivity, misunderstandings or negative reactions may occur [12]. ⑥ Insufficient skill development: Resident physicians need to master various technical skills in clinical practice, such as using laryngoscopes, nasal endoscopes, and otoscopes. Without effective skill training, it is challenging to improve medical competence and service quality [13].

After six months of training using a combination of CBL, the SEGUE framework, and incorporating special doctor-patient communication scenarios, there was a significant improvement in the SEGUE scores of the 120 resident physicians. Based on the analysis and summary, during the clinical teaching process, the instructors provided resident physicians with typical cases for discussion and analysis. By studying these cases, resident physicians gained an understanding of how to effectively communicate with patients using the SEGUE framework and acquired specific skills and techniques.The second round of SEGUE scores also demonstrated the significant effectiveness of the combined application of CBL and the SEGUE framework. Through case-based learning and discussions, resident physicians not only mastered basic medical knowledge and skills but also learned how to effectively communicate with patients. They learned how to listen to patients’ needs and concerns, ask clear questions, provide concise explanations, and reach consensus with patients.By combining CBL and the SEGUE framework, resident physicians can continuously improve their medical knowledge and doctor-patient communication skills through practical experiences. They can gain in-depth understanding of various diseases and clinical situations, enhance their clinical reasoning abilities, and improve their problem-solving skills. Simultaneously, resident physicians can communicate more effectively with patients, establish a good doctor-patient relationship, and improve patient treatment outcomes and satisfaction.

### Factors influencing the doctor-patient communication skills of resident physicians

In this study, different analyses were conducted based on gender, age, grade, educational background, and marital status. The results indicate that there were no significant differences in SEGUE scores among resident physicians of different genders and marital statuses. SEGUE scores were positively correlated with age. Different grades and educational backgrounds had a significant impact on SEGUE scores.In the initial evaluation, SEGUE scores increased with higher grades, and the second evaluation also showed that second- and third-year residents had significantly higher scores than first-year residents. In both evaluations, doctoral students had significantly higher SEGUE scores than master’s students and undergraduate students, while there was no significant difference in SEGUE scores between master’s students and undergraduate students.The analysis suggests that master’s students and undergraduate students received similar clinical practice guidance and theoretical training in otolaryngology before entering the specialized base. They were of similar ages during their residency training. As resident physicians accumulated clinical practice experience and training time, their doctor-patient communication skills gradually improved. Based on a certain level of clinical practice, resident physicians gradually mastered the process and content of doctor-patient communication, that is, knowing what to say and how to say it. This enables them to gradually convey information to patients that they may not be aware of, thereby increasing mutual trust and cooperation between doctors and patients [14].

### Reflection on the evaluation process of doctor-patient communication skills in resident physicians

Doctor-patient communication skills consist of basic language abilities as well as comprehensive communication skills, linguistic adaptability, and humanistic care abilities with the characteristics of the medical profession [15]. In modern society, doctors and patients are burdened with more complex factors such as economics, politics, culture, environment, and psychology. Therefore, medical practice requires a continuous strengthening of the thinking and behavior of doctor-patient communication [16]. In the new environment, healthcare professionals need the active participation of patients in both subjective and objective aspects of the medical process. Inquiry preparation, information gathering, and information provision all rely on the active cooperation of patients and their families. Regarding the current status of doctor-patient communication in otolaryngology resident physicians, in order to improve the learning outcomes and clinical practice abilities of resident physicians and facilitate effective communication, we can establish a teaching model centered around resident physicians. Resident physicians can participate in case analysis, diagnosis, treatment, and other processes, allowing them to solve practical problems through their own thinking and exploration. This approach not only enables resident physicians to acquire basic medical knowledge and skills but also helps them understand how to effectively communicate with patients. They learn how to listen to the needs and concerns of patients, how to ask precise questions, how to provide clear explanations, and how to reach consensus with patients.

One limitation of this study is the lack of a control group, who did not receive the combined-method training. The biggest difficulty preventing us to do so was that multiple centers were involved in our study. It was not plausible to take participants from some institutes as a control while the others as the treatment group as it would also create bias. On the other hand, it was unreasonable to divide participants from one single institute into different groups (the trained and untrained) since we were concerned that they might affect each other. However, to perform a more convincible study, we can design an experiment in the future with a larger sample size so that the participants can randomly divided into two groups in each clinical center, with the treatment group receiving the CBL-SEGUE Framework teaching method while the control receiving the traditional teaching method. But the interaction between them should be prohibited.

## Conclusion

Clinical teaching for otolaryngology resident physicians faces numerous challenges, requiring collaborative efforts from medical schools and teachers to explore better teaching methods and strategies. Combining the CBL approach with the SEGUE Framework proves to be a highly effective method for teaching and communication, enabling resident physicians to enhance their medical knowledge and improve their doctor-patient communication skills in the field of otolaryngology. Furthermore, it also enhances their self-learning abilities and self-management skills. Therefore, this approach is worth promoting and applying in other medical specialties.

## Data Availability

The datasets used and analysed during the current study are available from the corresponding author on reasonable request.
